# Factors Influencing Medical Students' Choice of Anesthesiology as a Future Specialty in Saudi Arabia

**DOI:** 10.7759/cureus.68028

**Published:** 2024-08-28

**Authors:** Awadh M Alharbi, Ahmed A Alsultan, Jawharah M Tirkistani, Abdullah A Alharbi, Maram H Asiri, Reema E Aloteibi, Reem F Bahakeem, Khalid N Bin Ghali, Omar S Al Misnid

**Affiliations:** 1 Medicine, Unaizah College of Medicine and Medical Sciences, Qassim University, Unaizah, SAU; 2 Medicine, Al-Ahsa Health Cluster, Al-Ahsa, SAU; 3 Medicine, Faculty of Medicine, Tanta University, Tanta, EGY; 4 Medicine, Faculty of Medicine, Umm Al-Qura University, Makkah, SAU; 5 Medicine, College of Medicine, King Khalid University, Abha, SAU; 6 Medicine, College of Medicine, King Saud bin Abdulaziz University for Health Sciences, Jeddah, SAU; 7 Anesthesiology, King Faisal Specialist Hospital and Research Centre, Riyadh, SAU; 8 Department of Emergency, Critical Care and Anesthesia, College of Medicine, Qassim University, Qassim, SAU

**Keywords:** career, choice, medical students, saudi arabia, anaesthesiology

## Abstract

Background

According to the American Society of Anesthesiologists (ASA), anesthesiologists are experts in administering anesthesia, pain management, and critical care medicine. In addition, they provide general perioperative care. Personal interests, career stability, reputation, income, and clinical rotation experience influence medical students' career choices. Studies show that anesthesiology remains one of the least popular specialties among Saudi medical students. Our study aims to determine the preference for anesthesiology among Saudi medical students and the factors influencing their career choice options.

Methodology

This cross-sectional study was implemented by distributing a self-administered verified survey.

Results

Our study included 532 medical students, predominantly female (n=344, 64.7%), aged 18-24 years (n=424, 79.7%), and Saudi nationals (n=508, 95.5%). Most were single (n=500, 94%) without children (n=522, 98.1%). A majority were not interested in anesthesiology as a future specialty (n=297, 55.8%), with some uncertainty (n=148, 27.8%) and a smaller interested group (n=87, 16.4%). Controllable lifestyle (n=294, 55.3%) and financial income (n=213, 40%) were critical factors for choosing anesthesiology as a specialty. Interest in another specialty (n=342, 64.3%) and stress (n=286, 53.8%) were significant opposing factors. Academic year significantly affected consideration (p<0.001), with second-year students showing the highest interest (61.8%) and sixth-year the lowest (24.8%). Other factors showed no significant association.

Conclusion

Our study reveals low interest in anesthesiology among Saudi medical students. A controllable lifestyle and financial income are key attractions. Interest declines significantly by the sixth year due to their interest in other specialties, lifestyle concerns, and stress.

## Introduction

According to the American Society of Anesthesiologists (ASA), anesthesiologists are experts in administering anesthesia, pain management, and critical care medicine. In addition, they provide general perioperative care. Anesthesiologists oversee the preoperative assessment of patients, create anesthetic treatment plans, deliver and monitor anesthesia during procedures, and optimize postoperative care. They also play a crucial role in providing essential care, managing acute and chronic pain, and performing clinical research and teaching to improve patient outcomes and safety [1]. Anesthesia is divided into three primary categories: general, regional, and monitored anesthesia care. From short and simple cases to lengthy and complicated surgeries, general anesthesia is frequently required, which causes unconsciousness. The use of anesthetics is not confined to anesthesiologists; a few medical specialists can give anesthesia, particularly local anesthesia, to some extent [2].

Undergraduate students in medical school have a wide range of specialties, including general surgery, internal medicine, orthopedic surgery, and anesthesiology. The student's interests, career stability, reputation, prestige, and income are just a few variables that studies have identified potentially influencing medical students' career choices [3]. Additionally, some research has revealed that knowing the specialties beforehand and being a senior student both significantly influence the students' choice. These show that clinical rotation impacts students' evaluation of specialization [4].

A recent local study was done on 248 students. Students were permitted to select several fields of interest, and the result is as follows: internal medicine (43.5%), surgery (36.7%), family medicine (27.4%), pediatrics (23.4%), obstetrics and gynecology (18.1%), radiology (14.5%), and anesthesiology (14.5%) [5]. Another stated that the least popular specialties were radiology (0.4%) and anesthesiology (0.8%). These findings conclude that anesthesiology remains one of the least popular specialties among medical students [2].

However, how students feel about anesthesiology as a potential career is still being determined as to which specializations they prefer and which local factors most influence their decisions. Determining this is crucial to modifying educational methods and raising student engagement levels. Fundamentally, the medical specialization that medical professionals choose as a career determines the future supply of doctors who will be accessible for the nation's healthcare services.

Umm Al-Qura University previously conducted a study on their students, and their results showed that most students (72.2%) are not interested in choosing anesthesiology as a future career [6]. However, due to the results being limited to a smaller population, the present study aims to determine collectively whether anesthesiology is a preferred specialty among medical students in Saudi Arabia and what factors may have helped or hindered their choice. This will address the shortage of anesthesiologists in the country, promote specialized training, and ultimately improve the quality of anesthesiology care in Saudi Arabia.

Therefore, the purpose of this study was to understand the attitude of medical students across Saudi Arabia toward anesthesiology as a specialty and to determine the common factors influencing their career choice options.

## Materials and methods

This study adopts a quantitative analytical observational cross-sectional design and was conducted among medical students at various universities across Saudi Arabia, including Umm Al-Qura University, King Khalid University, Qassim University, King Saud bin Abdulaziz University for Health Sciences, and King Abdulaziz University, from June 2023 to May 2024. Assuming a population size of 36,000 medical students enrolled in universities across Saudi Arabia, with a 95% confidence level, the required sample size was calculated using the following formula: n = (Z^2 * p * q) / d^2. Z is the desired confidence level. For a 95% confidence level, the Z-score was 1.96. p is the estimated proportion of medical students interested in anesthesiology as a career choice, and because there is no prior data provided, we assumed that it was 50%. Therefore, p was 0.5. q is the proportion of medical students not interested in anesthesiology as a career choice, so it was also 0.5. D is the desired level of precision, which was 0.05. Plugging these values into the formula, the estimated sample size was calculated to be 385 participants.

To account for potential non-response or incomplete data, the sample size was further adjusted to include 532 participants. This larger sample size would ensure a more robust and reliable data set, allowing us to draw more accurate conclusions about the factors influencing medical students' career preferences in Saudi Arabia, particularly regarding their interest in the field of anesthesiology.

A non-probability convenience sampling technique was employed for this study. Our inclusion criteria included all undergraduate medical students across Saudi Arabia who are in their second to sixth year of medical school, as well as medical interns who have just completed their academic years. The exclusion criteria included first-year medical students because they are in the preparatory year, which consists only of foundational sciences; medical students outside of Saudi Arabia; students from other healthcare specialties; and those who did not complete the survey. Data was collected using a questionnaire developed by researchers at Umm Al-Qura University [3], consisting of three main sections with a total of 17 questions. The first section focused on gathering demographic information about the participants, such as their age, gender, university affiliation, and year of study. The second section explored the medical students' level of interest in pursuing anesthesiology as a career choice. The third section investigated the various factors that influenced their overall career preferences and decision-making processes. The questionnaire was distributed online for efficient and anonymous data collection. A pilot study involving 25 students was conducted to determine the reliability of the questionnaire using Cronbach's alpha. The collected data was analyzed using SPSS version 26.0 (BM SPSS Statistics, Armonk, NY), applying descriptive statistics and inferential analyses such as Chi-square and Fisher's exact tests, to investigate the relationships between variables and determine the statistical significance of the findings. The significance level was set at a p-value of 0.05.

Ethical approval has been obtained from the Committee of Research Ethics at Qassim University (approval number: 23-60-02), and informed consent was secured from all participants, ensuring privacy and confidentiality.

## Results

Table 1 shows that our study included 532 medical students, of which the majority were female (n=344, 64.7%) compared to males (n=188, 35.3%). Most students are aged 18-24 (n=424, 79.7%), with a smaller group aged 25-34 (n=108, 20.3%). The majority are Saudi nationals (n=508, 95.5%), with a minor proportion being non-Saudi (n=24, 4.5%). Many students are single (n=500, 94%), while a few are married or divorced (n=32, 6%). Most do not have children (n=522, 98.1%), with only a small number having children (n=10, 1.9%). Students are from various universities, notably Umm Al-Qura University (n=120, 22.6%) and King Khalid University (n=106, 19.9%). Academic years range from second year (n=34, 6.4%) to intern (n=102, 19.2%), with a grade point average (GPA) distribution showing a considerable portion having a GPA of 4.5-5 (n=198, 37.2%).

**Table 1:** Sociodemographic profile of students (N=532) GPA: grade point average

		Number	Percentage (%)
Gender	Female	344	64.7
	Male	188	35.3
Age	18-24 years	424	79.7
	25-34 years	108	20.3
Nationality	Non-Saudi	24	4.5
	Saudi	508	95.5
Marital status	Single	500	94
	Married/divorced	32	6
Have children	No	522	98.1
	Yes	10	1.9
Universities	Umm Al-Qura University	120	22.6
	King Khalid University	106	19.9
	Qassim University	97	18.2
	King Saud bin Abdulaziz University for Health Sciences	60	11.3
	King Abdulaziz University	31	5.8
	Other universities	118	22.2
Academic year	2nd	34	6.4
	3rd	61	11.5
	4th	116	21.8
	5th	70	13.2
	6th	149	28
	Intern	102	19.2
GPA	<4 GPA	186	35
	4-4.5 GPA	128	24.1
	4.5-5 GPA	198	37.2

Table 2 shows the general attitude toward an anesthesiology career among the students, and it shows that most are not interested in choosing anesthesia as a future specialty (n=297, 55.8%). At the same time, some are uncertain (n=148, 27.8%), and a smaller group is interested (n=87, 16.4%). Most students did not have an elective rotation in anesthesia (n=458, 86.1%), whereas a minority did (n=74, 13.9%), with rotation durations varying as follows: two weeks (n=40, 7.5%), three weeks (n=19, 3.6%), one month (n=37, 7%), and more than one month (n=12, 2.3%). Many do not have relatives or friends in the anesthetic field (n=388, 72.9%), but many have connections in the medical field (n=457, 85.9%). While 59.8% (n=318) do not consider anesthesiology a future career, 40.2% (n=214) do. Over half have been involved in research (n=298, 56%).

**Table 2:** General attitude toward anesthesiology career (N=532)

		Number	Percentage (%)
Interested in choosing anesthesia as a future specialty?	No	297	55.8
	Maybe	148	27.8
	Yes	87	16.4
Did you have an elective rotation in anesthesia?	No	458	86.1
	Yes	74	13.9
If you have had a clinical rotation in anesthesia, how long was the rotation?	2 weeks	40	7.5
	3 weeks	19	3.6
	1 month	37	7
	>1 month	12	2.3
Do you have a relative or a friend in the anesthetic field?	No	388	72.9
	Yes	144	27.1
Do you have a relative or a friend in the medical field?	No	75	14.1
	Yes	457	85.9
Do you consider anesthesiology as a future career?	No	318	59.8
	Yes	214	40.2
Were you involved in any research work?	No	234	44
	Yes	298	56

**Figure 1:**
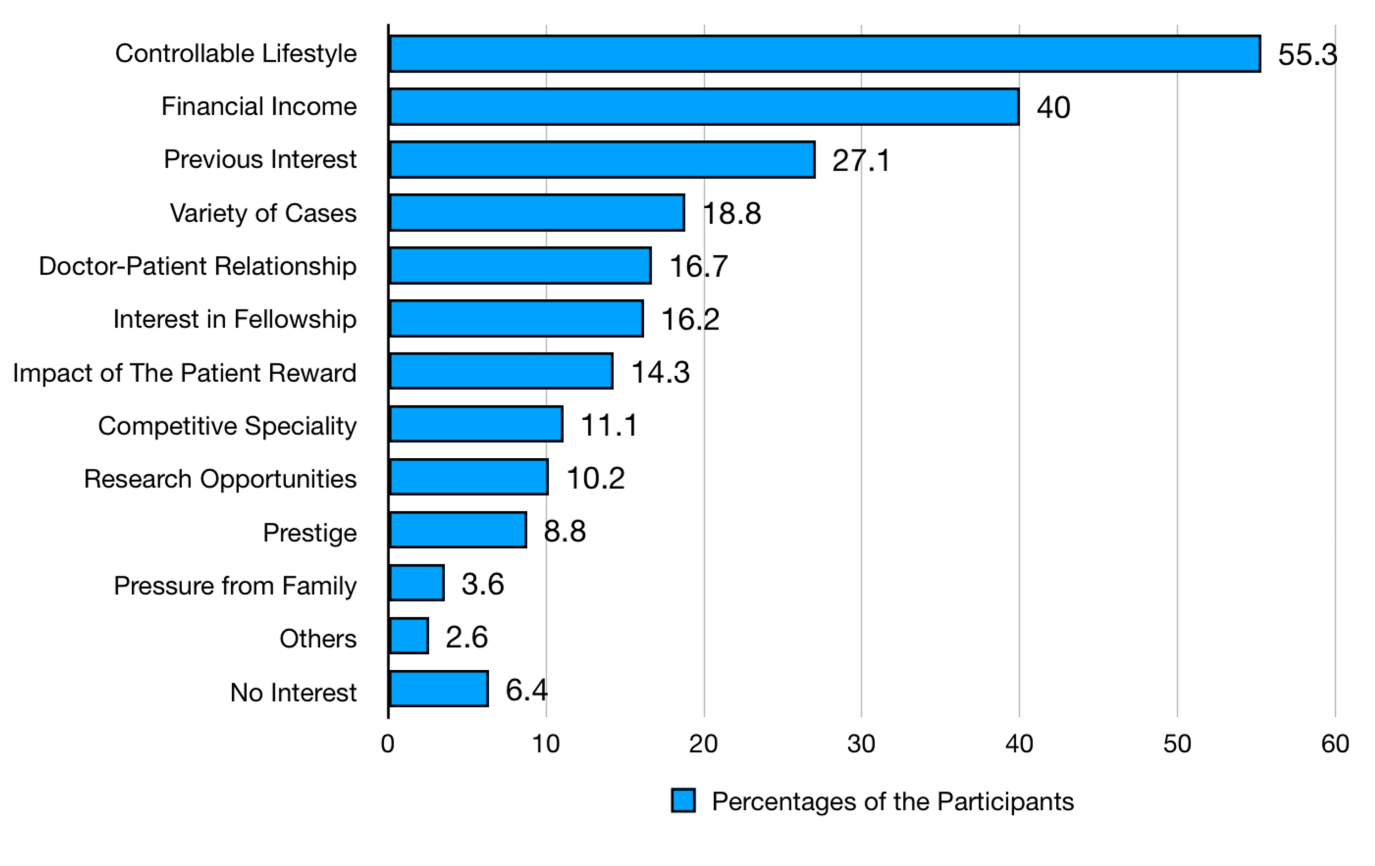
Students' driving factors to choose anesthesiology (N=532)

Figure 1 shows the driving factors influencing the choice of anesthesiology. The most significant factor is a controllable lifestyle (it refers to a specialty where the working hours are flexible, allowing for a better balance between social life and work life), with 55.3% (n=294) of students considering it necessary. Financial income is a substantial consideration for 40% (n=213) of the students. A previous interest in the specialty appeals to 27.1% (n=144), while 18.8% (n=100) value the variety of cases in anesthesiology. Doctor-patient relationships are essential for 16.7% (n=89), and interest in fellowship opportunities influences 16.2% (n=86). The impact of patient reward is a factor for 14.3% (n=76). Other considerations include the competitiveness of the specialty (n=59, 11.1%), research opportunities (n=54, 10.2%), prestige (n=47, 8.8%), and pressure from family (n=19, 3.6%). Additionally, 2.6% (n=14) cite other reasons, while 6.4% (n=34) are not interested in anesthesiology.

**Figure 2:**
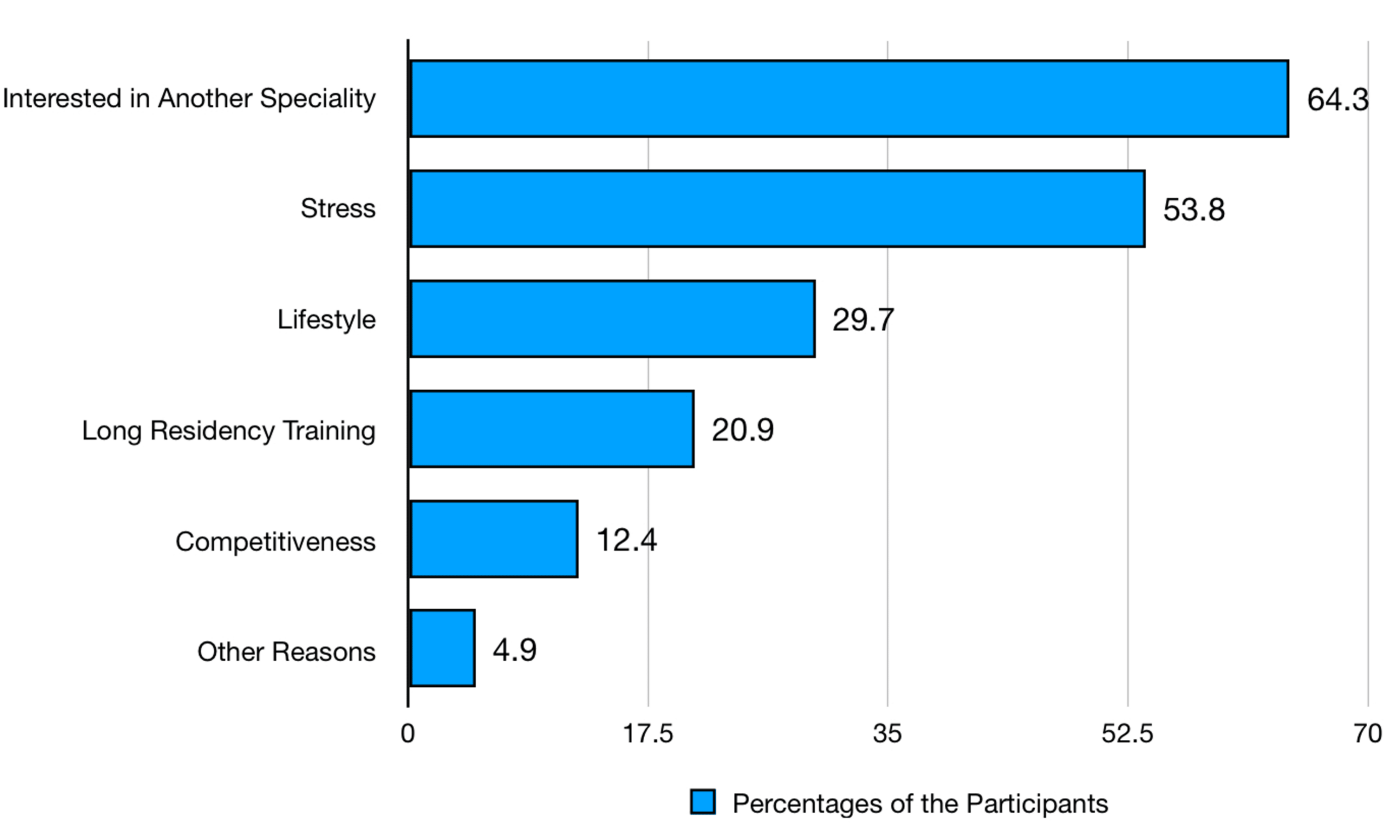
Students' deterring factors in choosing anesthesiology (N=532)

Figure 2 shows the factors restraining students from choosing anesthesiology as a specialty. The primary factor is interest in another specialty, cited by 64.3% (n=342) of the students. Stress is also a significant factor, affecting 53.8% (n=286). Lifestyle concerns deter 29.7% (n=158) of the students, while the length of residency training is a deterrent for 20.9% (n=111). The competitiveness of the specialty affects 12.4% (n=66), and other unspecified reasons are cited by 4.9% (n=26).

Table 3 shows the association between participants' consideration of anesthesiology as a future career and various factors. Gender did not show a significant association (p=0.407), with 41.6% (n=143) of females and 37.8% (n=71) of males considering anesthesiology. Age also showed no significant difference (p=0.449), with 41% (n=174) of 18-24-year-olds and 37% (n=40) of 25-34-year-olds being interested. Marital status was not significant (p=0.675), with 40% (n=200) of singles and 43.8% (n=14) of married students considering the specialty. Nationality was non-significant (p=0.154), with 54.2% (n=13) of non-Saudis and 39.6% (n=201) of Saudis being interested. Having children was also non-significant (p=0.533), with 50% (n=5) of those with children and 40% (n=209) of those without considering anesthesiology. Academic year showed a significant association (p<0.001), with the highest interest in the second year (61.8%, n=21) and the lowest in the sixth year (24.8%, n=37). The name of the college approached almost significance (p=0.091), with King Khalid University having the highest interest (48.1%, n=51). GPA did not show a significant association (p=0.163), with the highest interest in the 4-4.5 GPA range (43.8%, n=56) and the lowest in the 4.5-5 GPA range (34.3%, n=68).

**Table 3:** Association between participants' consideration of anesthesiology as a future career and different features (N=532) ^a^Chi-square test ^b^Fisher's exact test GPA: grade point average

			Consideration of anesthesiology as a future career		Significance value
			No	Yes	
Gender	Female	Number	201	143	0.407^a^
		Percentage (%)	58.4%	41.6%	
	Male	Number	117	71	
		Percentage (%)	62.2%	37.8%	
Age	18-24 years	Number	250	174	0.449^a^
		Percentage (%)	59%	41%	
	25-34 years	Number	68	40	
		Percentage (%)	63%	37%	
Marital status	Single	Number	300	200	0.675^a^
		Percentage (%)	60%	40%	
	Married	Number	18	14	
		Percentage (%)	56.3%	43.8%	
Nationality	Non-Saudi	Number	11	13	0.154^a^
		Percentage (%)	45.8%	54.2%	
	Saudi	Number	307	201	
		Percentage (%)	60.4%	39.6%	
Do you have children?	No	Number	313	209	0.533^b^
		Percentage (%)	60%	40%	
	Yes	Number	5	5	
		Percentage (%)	50%	50%	
Academic year	2nd	Number	13	21	<0.001^a^
		Percentage (%)	38.2%	61.8%	
	3rd	Number	28	33	
		Percentage (%)	45.9%	54.1%	
	4th	Number	66	50	
		Percentage (%)	56.9%	43.1%	
	5th	Number	31	39	
		Percentage (%)	44.3%	55.7%	
	6th	Number	112	37	
		Percentage (%)	75.2%	24.8%	
	Internship	Number	68	34	
		Percentage (%)	66.7%	33.3%	
Name of college	Umm Al-Qura University	Number	81	39	0.091^a^
		Percentage (%)	67.5%	32.5%	
	King Khalid University	Number	55	51	
		Percentage (%)	51.9%	48.1%	
	Qassim University	Number	57	40	
		Percentage (%)	58.8%	41.2%	
	King Saud bin Abdulaziz University for Health Sciences	Number	42	18	
		Percentage (%)	70%	30%	
	King Abdulaziz University	Number	18	13	
		Percentage (%)	58.1%	41.9%	
	Other	Number	65	53	
		Percentage (%)	55.1%	44.9%	
GPA	<4	Number	108	78	0.163^a^
		Percentage (%)	58.1%	41.9%	
	4-4.5	Number	72	56	
		Percentage (%)	56.3%	43.8%	
	4.5-5	Number	130	68	
		Percentage (%)	65.7%	34.3%	

## Discussion

The duties of anesthesiologists include administering anesthetics, monitoring hemodynamics, performing preoperative evaluations, coming up with treatment plans, preserving anesthesia during procedures, ensuring postoperative care is provided, and managing acute and chronic pain [7]. Sawan et al. (2023) [8] show that several factors can influence medical students' choice of specialty, including personal interest, job security, financial stability, gender, and family circumstances. Clinical rotations significantly impact their specialty preferences [9]. A local study by Alrajban et al. (2024) [10] showed low interest (14.5%) in anesthesiology among students. Our study aimed to explore Saudi medical students' preferences for anesthesiology and the factors influencing their choices, which are essential for adjusting educational strategies and ensuring future healthcare workforce needs.

According to our study, most medical students (55.8%, n=297) are not interested in anesthesiology as a future career (Table 2). This finding aligns with previous studies conducted in various countries, where anesthesiology is often perceived as less attractive than other specialties such as surgery or internal medicine. Prior surveys by Arefayne et al. (2022) [11] and Bhattarai et al. (2012) [12] have shown that among medical doctors and students, anesthesiology has not been shown as an attractive and demanding specialty that most physicians do not want to specialize in the field. The group still determines their specialty (27.8%, n=148), and those interested (16.4%, n=87) reflect the need for better exposure and education about the field during medical training (Table 2). The high percentage of students without elective rotations in anesthesiology (86.1%, n=458) underscores a potential area for curriculum enhancement (Table 2). Studies have shown that increased exposure to anesthesiology through clinical rotations can significantly improve students' perceptions and interest in the specialty. Galway (2010) [13] shows that 96% of the students surveyed stated that the anesthesia clerkship increased their desire to pursue a career in the field.

Moreover, there are various driving factors for choosing anesthesiology, which include the importance of a controllable lifestyle (55.3%, n=294) (Figure 1). This aligns with global trends where lifestyle considerations, including work-life balance and flexible working hours, play a critical role in specialty selection. Levaillant et al. (2020) [14] show that the main factors influencing the choice of specialty were lifestyle, work-life balance, and discipline interest. Financial income (40%, n=213) and a previous interest in the specialty (27.1%, n=144) also emerged as significant motivators; financial prospects and pre-existing interests strongly influence career decisions (Figure 1). Nguyen and Bounds (2019) [15] show that medical students appear to select their specialties for economic reasons.

Moreover, Karthik et al. (2023) [16] show that several factors impact specialty decisions, including academic interests. Interestingly, factors such as doctor-patient relationships (16.7%, n=89) and interest in fellowship opportunities (16.2%, n=86) were less frequently cited (Figure 1). This may reflect a perception that anesthesiology offers fewer opportunities for patient interaction and post-graduate specialization than other fields. The impact of patient reward (14.3%, n=76) and prestige (8.8%, n=47) were also lower (Figure 1), possibly due to a general underestimation of anesthesiologists' critical role in patient outcomes and surgical success. Enhancing awareness of these aspects could shift perceptions.

Moreover, the primary opposing factor for choosing anesthesiology was interest in another specialty (64.3%, n=342), a common theme in medical education literature. Stress (53.8%, n=286) and lifestyle concerns (29.7%, n=158) were significant barriers (Figure 2), reflecting the demanding nature of the specialty and its impact on personal life. These findings are supported by studies from North America and Europe, where stress and work-life balance are consistently cited as major concerns among medical students considering anesthesiology. Shams et al. (2015) [17] show that lifestyle issues are often cited as essential in selecting a specialty for residency training, and anesthesiology is frequently included in lists of specialties associated with favorable lifestyles. The length of residency training (20.9%, n=111) and competitiveness of the specialty (12.4%, n=66) were also notable deterrents (Figure 2). This aligns with the literature suggesting that lengthy training programs and competitive entry requirements can dissuade students from pursuing certain specialties. Madden et al. (2023) [18] show that the negative predictors of career choice were a lack of interest in the area, perceived workload, and duration of training schemes. Addressing these issues through targeted mentorship programs and providing clearer career pathways could mitigate some of these concerns.

Moreover, our study found no significant association between gender and consideration of anesthesiology as a career (p=0.407) (Table 3), contrasting with some studies indicating gender differences in specialty preferences. Carter et al. (2024) [19] show that gender inequity remains an issue in anesthesiology despite increasing numbers of women training and achieving fellowship in the specialty. Women are under-represented in all areas of anesthetic research, academia, and leadership. Age, marital status, nationality, and having children also showed no significant associations (Table 3), suggesting that interest in anesthesiology is relatively uniform across these demographics in our sample. However, the academic year showed a significant association (p<0.001), with the highest interest observed in the second year and the lowest in the sixth year. This could be due to the evolving interests and increased exposure to different specialties as students progress through their medical education. Early exposure to anesthesiology and positive experiences during clinical rotations could sustain interest throughout medical school.

The near-significant association with the college name (p=0.091) suggests institutional differences in how anesthesiology is perceived and taught. King Khalid University had the highest interest (48.1%, n=51), possibly reflecting a more robust anesthesiology program or more significant faculty influence. This finding is consistent with studies indicating that the quality of faculty and curriculum can significantly impact specialty choice [20].

Limitations

Our study has several limitations, including potential selection bias, and self-reported data may introduce response bias. Generalizability may be limited to Saudi medical students. Additionally, factors not explored in the study, such as cultural influences or faculty perceptions, could impact career choices. Further research with larger, more diverse samples and longitudinal designs is warranted to address these limitations.

## Conclusions

Our study provides valuable insights into the attitudes and factors influencing medical students in Saudi Arabia toward anesthesiology. While controllable lifestyle and financial income are strong motivators, stress, lifestyle concerns, and interest in other specialties are significant deterrents. The lack of significant associations with most sociodemographic factors suggests a relatively uniform perception of anesthesiology among different groups of students. However, the substantial impact in the academic year highlights the importance of early and sustained exposure to the specialty. Future efforts should focus on enhancing the visibility and appeal of anesthesiology through targeted educational interventions, mentorship programs, and addressing perceived barriers. Further research is needed to explore these findings in different cultural and academic contexts to develop more comprehensive strategies for attracting students to anesthesiology.
